# Vitamin D_3_ Nutritional Status Affects Gut Health of *Salmonella*-Challenged Laying Hens

**DOI:** 10.3389/fnut.2022.888580

**Published:** 2022-05-10

**Authors:** Fangshen Guo, Yanqiang Geng, Waseem Abbas, Wenrui Zhen, Shuiqing Wang, Yuechuan Huang, Yuming Guo, Qiugang Ma, Zhong Wang

**Affiliations:** State Key Laboratory of Animal Nutrition, College of Animal Science and Technology, China Agricultural University, Beijing, China

**Keywords:** Vitamin D, *Salmonella*, intestinal health, gut microbiota, laying hen

## Abstract

*Salmonella enterica* serovar Enteritidis (SE) is one of the most common pathogens associated with poultry health and foodborne Salmonellosis worldwide. The gut plays a pivotal role in inhibiting SE transintestinal transmission and contaminating poultry products. The nutritional status of vitamin D (VD) is involved in gut health apart from bone health. However, the impact of VD_3_ nutritional status on the gut health of *Salmonella*-challenged hens is rarely investigated. This study investigated the impact and possible mechanisms of VD_3_ nutritional status on the gut health of hens challenged with SE. Hens were fed basal diets with either 0 (deficient) or 3000 IU (sufficient) VD_3_/kg of diet, respectively. After 10 weeks of feeding, half of the hens were orally inoculated with either SE (1 × 10^9^ CFU /bird). Results indicated that VD_3_ sufficiency reversed the disruptive effects on the laying performance of hens caused by *Salmonella* challenge or VD_3_ insufficiency by promoting VD_3_ metabolism. In addition, VD_3_ sufficiency ameliorated gut injury induced by either *Salmonella* or VD_3_ deficiency, shown by reducing *Salmonella* load and histopathological scores, suppressing TLR4-mediated inflammatory responses, and increasing expression of TJs along with decreasing pro-apoptotic protein expression and the number of TUNEL-positive cells in the jejunum. Besides, VD_3_ enriched the abundance of probiotics, such as *Lactobacillus* and *Bacilli*, and restored the balance of gut microflora. Collectively, dietary VD_3_ sufficient supplementation could alleviate *Salmonella* or VD_3_ deficiency-induced intestinal damage of hens *via* modulating intestinal immune, barrier function, apoptosis along with gut microbiota composition, revealing that VD_3_ could act as a novel nutritional strategy defending *Salmonella* invasion in hens.

## Introduction

*Salmonella enterica* serovar Enteritidis (*S*. Enteritidis, SE), one of the most prevalent serotypes of *Salmonella*, is a common foodborne pathogen that enters the human food chain through animal products, particularly raw poultry products resulting in human salmonellosis worldwide (1). *S. Enteritidis* infection in chicks can result in asymptomatic cecal colonization, gut dysbiosis, intestinal mild inflammation (2, 3), and gut barrier damage along with the invasion of internal organs and poultry carcasses (4). In addition, its infection on hens would induce SE transintestinal transmission and colonizing reproductive tissues (ovaries, oviduct, and testis), and persistent shedding of *S. enteritidis* in feces and eggs laid by infected hens (5). The report revealed that more than 90% of human salmonellosis was related to the *Salmonella* contamination in eggs and chicken, of which SE is the most frequent serotype inducing human salmonellosis (6, 7). Thus, infected chickens and contaminated poultry products are one of the most important carriers of SE threatening human health, which was considered to be one of the major sources of human salmonellosis worldwide (8). Therefore, reducing *S*. Enteritidis colonization and invasion in the intestinal tract of chickens, maintaining gut barrier integrity and intestinal microbial flora balance, along with improving intestinal mucosal immune defense ability would prevent *Salmonella* to invade the internal organs, ovaries, oviduct, and eggs and help minimize the contamination of poultry products and salmonellosis in humans.

Contamination of poultry products caused by *Salmonella* infection occurred frequently in the poultry industry. Biosecurity management and pharmaceutical approaches, such as vaccination, competitive exclusion, and antibiotic treatments, can help reduce the *S*. Enteritidis burden in poultry (2, 9). However, the use of in-feed growth-promoting antibiotics was gradually banned in recent decades, and the use of therapeutic antibiotics in aquaculture is also been reduced due to the risks associated with antibiotics use in some countries or areas (10). Supplemented with non-antibiotic and non-nutritional additives, such as probiotics, prebiotics, organic acids, short and medium-chain fatty acids, essential oils, nitro-compounds, and bacteriophages, in feed or drinking water to control *Salmonella* infection become popular on farms. Methods above were reported to help hosts resistance against *Salmonella* infection *via* enhancing innate immune functions and inhibiting inflammatory responses, modulating the balance of intestinal microbiota and competitively eliminating *Salmonella* colonization, repairing damaged intestinal barrier structure, killing directly or inhibiting indirectly the colonization, growth, and organ invasion of *Salmonella* ultimately (11–13). Meanwhile, dietary interventions with fortified nutrients (vitamins, minerals) were also suggested to enhance innate immune response and disease resistance against *Salmonella* infection in hens (14).

Vitamin D (VD) is a fat-soluble vitamin and pleiotropic hormone that has conventional roles in modulating calcium and phosphate absorption in the intestine, sustaining the homeostasis of calcium and phosphate, and bone mineralization along with skeletal health (15). Interestingly, in the past decades, accumulating data has shown that VD possessed several extra-skeletal effects, such as immunomodulatory, anti-inflammatory, anti-viral, anti-oxidative, anti-allergy, anti-apoptosis actions, and cancer prevention, through VDR-mediated regulation on gene expressions, mainly because VDR and/or VD 1α-hydroxylase were found to express in various tissues and cells (16, 17). Also, growing pieces of evidence from mammals' experiments have demonstrated that VD and VDR have an important role in maintaining gut homeostasis through enhancing the intestinal mucosal barrier integrity, sensing gut microbiota, preventing pathogens colonization and invasion, modulating innate and acquired immunity in the gut together with suppressing gut inflammation (18). VD deficiency or lack of VDR results in intestinal microbiota dysbiosis, inflammation, and barrier impairment along with increased susceptibility to infection and mucosal damage as well as disease exacerbation (19). It was suggested that a VD supplement could suppress intestinal mucosal inflammation and ameliorate intestinal injury by promoting antimicrobial peptides expressions, maintaining gut microflora eubiosis, along with enhancing tight junction (TJ) proteins expression (20, 21). Ulteriorly, supplementation with VD_3_ or its active form has been a beneficial approach to control enteric pathogens, such as *Helicobacter pylori, Clostridium difficile, Salmonella*, and *Citrobacter rodentium* infection and inflammatory bowel disease in mammals (22). Thus, the maintenance of an adequate VD status seems to provide a great preventive health potential.

Vitamin D is also one of the key dietary factors for hens. The essentiality of VD in hen feed is particularly significant for profitable egg and meat production in the modern poultry industry where the birds are raised indoors (23). VD_3_ has traditionally been used as a major source of VD by the poultry industry. Official recommendations the nutrient requirement for normal egg production and shell quality is 500 IU of cholecalciferol/kg feed. In China, the recommended common levels of supplemented VD_3_ specified by the China National Standardization Management Committee (NY/T33-2004) is 1,600 IU/kg feed for commercial laying hens, 2,000 IU/kg in diets for the commercial egg breeders, while 3,000–5,000 IU/kg diet is recommended by commercial producers. When laying hens are fed a diet deficient or completely devoid of VD_3_, the signs of deficiency are characterized by thin eggshells, repressed egg production, cracked eggshells, elevated mortality of birds, and low Ca and P utilization alone with skeletal diseases, and welfare issues (23). It was demonstrated VD deficiency aggravated the CD8+ T cell deficiency, decreased cell-mediated immune responses, and impaired macrophages development, resulting in higher susceptibility to pathogen infection and ultimately decreased growth performance in poultry (24). Additionally, previous studies showed that VD_3_ or its activated form (25-hydroxyvitamin D3) could serve as prophylactic ways to control pathogens infection and alleviate negative effects caused by pathogens, such as *Salmonella* and *Coccidia* infection in broilers (25).

Although, the above research showed that VD_3_ could serve as a prophylactic or therapeutic way to control enteric inflammation and pathogens infection in humans and broilers. However, to date, there are few studies investigating the influences of VD_3_ nutritional status on gut health of the *Salmonella* enteritis–challenged hens. Therefore, the objective of this study was to evaluate the impact of VD_3_ nutritional status on gut health of the *Salmonella*-challenged laying hens by determining intestinal *Salmonella* load, intestinal pathological changes, barrier function, apoptotic status, and immune function as well as the gut microbiome, to reveal the association between VD_3_ status and *Salmonella* resistance of laying hens, providing a feasible nutritional approach to mitigate the deleterious impacts on hens health caused by *Salmonella* infection and reduce the risk of *Salmonella* pollution on poultry products.

## Materials and Methods

### Animal Ethics Statement Experimental

All the procedures implemented in this study were approved by the China Agricultural University Animal Care and Use Committee (statement no. CAU20170601-2). All efforts were made to minimize animal suffering.

### Animals, Diets, and Treatments

A total of 144 *Salmonella*-negative Jinghong-1 strain layers at 32 weeks of age (a China local layer breed) were selected and randomly assigned into two groups, with 12 replicates per group and six birds per replicate. Hens were fed a corn-soybean-based meal mash diet without supplemental cholecalciferol (0 IU cholecalciferol /kg diet; VD_3_-deficient group) and the basal diet contained 3,000 IU cholecalciferol per kg diet (VD_3_-sufficient group) for 10 weeks, respectively. After 10 weeks of pre-feed, half of the hens in each group were orally gavaged with *Salmonella* enterica (1 × 10^9^ CFU /bird), whereas another half of the birds with sterile medium. All hens were re-assigned to 4 groups based on VD_3_ and *Salmonella* treatment, containing hens fed a VD-deficient diet with or without *Salmonella* challenge (group VD0S and VD0), and hens fed the VD-sufficient groups challenged with or without *Salmonella* (group VD3000S and VD3000). All infected hens (group VD0S and VD3000S) were reared in a separate room to avoid cross-infection. The basal diet was formulated to meet or exceeded the Chinese Feeding Standard of Chickens (NY/T33-2004) and nutrient requirements of laying hens except for VD_3_. The basal composition and nutrient levels of the diet are presented in Table 1. Hens were housed in climate-controlled experimental layer rooms and kept in 3-layer complete ladder cages at three birds per cage (40 cm × 45 cm × 45 cm) equipped with water nipples, respectively. All birds were fed *ad libitum* and allowed to access water freely throughout the entire experimental period. The lighting schedule was maintained on a cycle of 16 h light and 8 h dark. The average house temperature in the chicken house was 24.5 ± 0.5°C. The infection trial lasted for 2 weeks.

**Table 1:** Composition and nutrient levels of the basal diet.

**Items**	**Content**	**Items**	**Content**
Ingredient		Nutrient levels^c^	
Corn	66.45	Crude Protein	15.52
Soybean meal	22.80	Metabolic energy (MJ/kg)	11.30
Limestone	8.20	Calcicum	3.60
Calcium hydrogen phosphate	1.70	Available phosphorus	0.39
Sodium chloride	0.30	Lysine	0.75
DL-methionine	0.12	Methionine	0.37
Choline chloride	0.10	Met+Cys	0.64
Vitamin Premix^a^	0.03	Tryptophane	0.18
Trace elements^b^	0.30	Threonine	0.57
		Lysine	0.64
Total	100.00	Digestible Methionine	0.35

a *Provided per kilogram of diet: vitamin A, 6 000 IU; vitamin E, 21 IU; vitamin E, 21 IU; vitamin K_3_, 4.2 mg; vitamin B_1_, 3 mg; vitamin B_2_, 10.2 mg; folic acid, 0.9 mg; pantothenic acid, 15 mg; niacin, 45 mg; vitamin B_6_, 5.4 mg; vitamin B_12_, 24 μg; biotin, 0.15 mg*.

b *Mineral premix provided per kilogram of complete diet: copper, 6.8 mg; iron, 66 mg; zinc, 83 mg; manganese, 80 mg; iodine, 1 mg; se 0.3 mg. VD_3_ added alone. Prepared with a small mixing machine after premix mixing*.

*High performance liquid chromatography to detect the actual content, and the calculation of the value of the match before the feed preparation*.

c *Calculated nutrient levels*.

### Productive Performance

The egg number and total egg weight of each replicate pen were recorded daily, and the feed intake of each replicate was determined weekly. The laying rate, average egg weight, average feed intake, and feed to egg ratio were calculated before (from 1 to 10 weeks) and after SE infection (from 11 to 12 weeks). Hens were monitored twice daily for clinical expression of disease. Mortalities were recorded daily for 2 weeks post-SE infection.

### *Salmonella* Inoculation and Challenge

*Salmonella* enterica strain CVCC3377 was inoculated on Xylose Lysine Deoxycholate (XLD) Agar at 37°C for 24 h. The single colonies were selected and inoculated into Luria-Bertani (LB) broth overnight at 37°C with shaking. Inoculation dose dilutions of SE were made based on a concentration estimated from the optical density at 600 nm applied to an SE growth curve regression equation. Actual SE inoculation doses were then confirmed by serial plate dilutions of the inoculum.

### Sample Collection

On 7 days post-infection (dpi), one bird per replicate was randomly selected, blood samples were collected *via* wing venipuncture and clotted at room temperature, and then centrifuged at 3,000 × g for 5 min at 4°C to collect serum for calcium and 25-OH-VD measure. After the blood collection, all birds were killed by cervical dislocation, the middle segments of the jejunum (approximately 1 cm) were cut off and gently rinsed with ice-cold sterile saline to remove internal digesta, and then immediately frozen with liquid nitrogen and stored at −80°C for gene and protein expression determination. Subsequently, the distal jejunum segments were fixed in 4% paraformaldehyde immediately for H&E staining and immunohistochemistry analysis. Cecal digesta samples were put into sterile tubes, snap-frozen in liquid nitrogen, and transferred to −80°C for DNA extraction and gut microbiome analysis.

### Serum Calcium and 25-Hydroxycholecalciferol

Serum calcium content was measured using commercial colorimetric assay kits (Nanjing Jiancheng Bioengineering Institute, Nanjing, China). Serum 25-OH-VD concentrations were determined by ultrahigh-performance liquid chromatography–tandem mass spectrometry as previously described (26). A standard curve was obtained using dilutions of a 25-OH-VD standard (Iso Sciences, USA).

### *Salmonella* Loads in the Cecum

The number of *Salmonella* cells in the cecal content was analyzed using the plate pouring method as previously described (3). Bacterial concentration was expressed in colony-forming units (lg CFU/g of content).

### Histopathological Observation

The fixed jejunum tissues were processed in graded ethanol, then embedded in paraffin wax, cut into 5 μm thickness slides using a microtome (Leica Microsystems K.K., Tokyo, Japan), and stained with H&E. The slides were scanned (Leica Aperio CS2, Germany) for histopathological analysis at 200 × magnification using an Apeio Image Scope (Version 12.0.1; Leica Biosystems, Buffalo Grove, USA). Histopathological scoring refers to a standard described previously (27). The combined pathological score for jejunum tissue specimen was determined as the sum of following scores: (1) Inflammation (score 0-3): 0 = no inflammatory-cell infiltration; 1 = slight inflammatory-cell infiltration; 2 = moderate inflammatory-cell infiltration; 3 = severe inflammatory-cell infiltration. (2) Extent of lesions (score 0-3): 0 = No lesion; 1 = Lesion in the mucosal layer; 2 = Lesion in the mucosal layer and submucosa; 3 = Transparent cell wall. (3) Crypt Damage (score 0-4): 0 = No lesion in crypt; 1 = 1/3 crypt lesion; 2 = 2/3 crypt lesion; 3 = only the epithelial surface was intact; 4 = Crypt and epithelium not visible. It ranges between 0 and 10 arbitrary units and covers the levels of inflammation in the intestine.

### Terminal Deoxynucleotidyl Transferase dUTP Nick End Labeling (TUNEL) Staining

The apoptotic cells in the jejunum tissues were detected using the terminal deoxynucleotidyl transferase dUTP nick end labeling (TUNEL) assay kit (Roche Diagnostics GmbH, Mannheim, USA) in accordance with the manufacturer's protocol. Briefly, sections (5 μm) were deparaffinized in xylene, rehydrated in decreasing concentrations of ethanol, boiled in Citra buffer solution for 10 min, and digested in 0.5% pepsin for 60 min at 37°C, before endogenous peroxidase was quenched in 3% hydrogen peroxide following incubating with proteinase K. The slides were incubated with the terminal deoxynucleotidyl transferase (TdT)/digoxin-dUTP mixture for 2 h at 37°C. Following washes with TBS three times, the slides were enclosed with anti-digoxin-biotin conjugated antibody diluted 1:100 in blocking reagent and incubated for 30 min at 37°C. Subsequently, the sections were incubated with streptavidin-biotin complex (SABC) for 1 h at 37°C. Apoptotic cells were detected by a microscope (80i, Nikon Eclipse, Tokyo, Japan) after incubation in the 3,3′-diaminobenzidine (DAB) chromogen for approximately 6 min and slides were counterstained with hematoxylin. The integrated optical density (IOD) of TUNEL-positive cells was assessed by a digital microscope camera system (Nikon DS-Ri1, Japan) and Image-Pro Plus 6.0 software (Media Cybernetics, Inc., MD, USA) image analysis software.

### Quantitative Real-Time PCR

Total RNA was extracted from the jejunum samples using Trizol reagent (Invitrogen Life Technologies, Carlsbad, CA, USA) according to the manufacturer's instructions. The concentration and purity of total RNA were determined using a NanoDrop-2000 spectrophotometer (Thermo Fisher Scientific, Waltham, MA, USA). Then, complementary DNA (cDNA) was synthesized using Primer Script™ RT reagent Kit (Takara) according to the manufacturer's instructions. Using the synthesized cDNA as a template, RT-qPCR was performed in Applied Biosystems' 7,500 Fast Real-Time PCR System81iUSA), with SYBR Premix Ex Taq TMQ20 kit (Takara Biotechnology Co. Ltd., Dalian, China) in accordance with the manufacturer's guidelines. Relative mRNA levels were calculated using the 2 ^−Δ*ΔCT*^ method and normalized with β-actin. Relative expressions for target genes were calculated using that of the VD-supplemented but without the SE infection group as the reference (set to 1). The primer sequences used in this study were designed and synthesized by Sangon (China) and listed in Supplementary Tables 1, 2.

### Western Blot

About 0.2 g of frozen jejunal mucosa was homogenized and lysed in 1 mL of ice-cold radioimmunoprecipitation assay (RIPA) lysis buffer containing 10 μL PMSF [50 mmoL/L Tris-HCl (pH 7.4), 150 mmoL/L NaCl, 1% nondiet P (NP)-40,.1% SDS, 1.0 mmoL/L PMSF, 1 mmoL/L Na_3_VO_4_, 1 mmoL/L sodium fluoride (NaF), and protease and phosphatase inhibitor cocktail] (P1045, Beyotime Biotechnology, Co., Ltd., Beijing). The homogenate lysate was centrifuged at 12,000 × g for 15 min at 4°C to remove cellular debris, and then the supernatant was collected for total protein determination using a BCA protein assay kit (BTYA0301, Biodragon Immunotechnologies Co., Ltd., Beijing, China). Equal amounts of extracted protein (40 μg) were subjected to electrophoresis on 8, 10, or 12% SDS-PAGE gels (Tris-glycine-sodium dodecyl sulfate-polyacrylamide gel electrophoresis), and then transferred onto methanol presoaked polyvinylidene difluoride (PVDF) membranes (IPVH000101, Millipore, USA). The membranes were inoculated with 3% skimmed-milk solution in Tris-buffered saline containing 0.05% Tween-20 (TBST blocking solution) at 37°C for 1 h, and then were incubated with primary antibodies. Rabbit anti-mouse polyclonal primary antibodies against chicken B cell lymphoma (Bcl-2) and Fas were purchased from Wanlei Biotech Co., Ltd. (Shenyang, China), and p53, Caspase-3, and glyceraldehyde 3-phosphate dehydrogenase (GAPDH), were purchased from Suzhou Biodragon Immunotechnology Co. Ltd. (Suzhou, China, Supplementary Table 3). Rabbit anti-chicken polyclonal primary antibodies against chicken Claudin-1, Claudin-4, Occludin, and ZO-1 recombinant protein antigens were prepared in our laboratory (patents were being authorized). And then membranes were washed an additional three times with TBST and incubated with secondary goat anti-rabbit horseradish-peroxidase (HRP)-conjugated IgG (BF-3008, Biodragon Immunotechnologies, Co., Ltd., Beijing, China) at room temperature for 3 h. The filters were then stripped and reprobed with antibodies to total glyceraldehyde 3-phosphate dehydrogenase (GAPDH, YM 3029, immunoway, USA) to demonstrate equal loading. After incubation, the chemiluminescence signal was detected by using the enhanced/super ECL chemiluminescent kit (BF06053, Biodragon Immunotechnologies Co., Ltd., Beijing, China) and a luminescence imager (Tanon 5200, Tanon Science & Technology Co., Ltd., Shanghai, China). Detection and quantification of protein band density were determined using image-J (version 1.50i) software. The results are expressed as relative intensity compared to those of the normal controls.

### 16s rDNA Sequencing and Data Processing of Gut Bacteria

Genomic DNA of cecal digesta at 7 dpi was extracted using PowerSoil^®^ DNA Isolation Kit (ANBIOSCI Tech Ltd., USA) according to the manufacturer's instructions. The concentration, purity, and integrity of the isolated genomic DNA were measured using a NanoDrop ND-1,000 (Thermo Scientific, Wilmington, DE), and agarose gel electrophoresis, respectively. The qualified genomic DNA was used as a template for the V3~V4 region of bacterial 16S rRNA gene amplification with barcoded primer pair 338F: 5′-ACTCCTACGGGAGGCAGCA-3′ and 806R: 5′-GGACTACHVGGGTWTCTAAT-3′. Amplicon libraries were constructed and sequenced on Illumina HiSeq 2,500 platform (Illumina, San Diego, USA) at Biomarker Technologies Co. Ltd. (Beijing, China). The sequencing data were merged using FLASH (version 1.2.11) to get raw tags. Raw tags were subjected to filtration (Trimmomatic, version.33) and chimera sequences removed (UCHIME, version 8.1) to obtain effective tags. UCLUST was used to cluster effective tags into operational taxonomic units (OTUs) at a similarity level of 97% with Quantitative Insights into Microbial Ecology (QIIME) software (version 1.8.0) (28). Afterward, based on the Silva taxonomic database, OTUs were annotated. Venn diagram, Rarefaction curve, and bacteria relative abundance were created with R software (version 2.15.3). Alpha diversity, including ACE, Chao 1, Simpson, and Shannon index, was investigated by Mothur (vision 1.30), and the significance of these items was determined using the Mann-Whitney U test. Beta diversity was calculated from a binary-jaccard distance (PERMANOVA/ANOSIM analysis) in QIIME software. Two-sided Student's *t*-test was used to determine the significance of the differences between groups. Line Discriminant Analysis (LDA) Effect Size (LEfSe, http://huttenhower.sph.harvard.edu/lefse/) tool was used to determine statistically different biomarkers between groups (LDA value = 4) based on the taxonomic files obtained from the QIIME analysis (29). The raw sequences used in our study had been uploaded at the Sequence Read Archive of the National Center for Biotechnology Information, with the study accession number PRJNA759703. The functions of the cecum metagenomes were predicted using Phylogenetic investigation of communities by reconstruction of unobserved states (PICRUSt) analysis based on high-quality sequences (30).

### Statistical Analysis

All results are displayed as means ± SEM. For the normally distributed data (serum calcium and 25-OH-VD concentration, productive performance, intestinal histopathological scores, cecal *Salmonella* load, intestinal PCNA-positive and TUNEL-positive cell numbers, relative mRNA expression, protein expression), the main effect and interaction effects were analyzed using the general linear model (GLM) procedure in SPSS 19.0 (SPSS Inc, Chicago, Illinois, USA). The one-way ANOVA and multiple comparisons were performed when interactive effects differed significantly. α-diversity and β-diversity were analyzed using the Mann-Whitney U test. The relative abundance of microorganisms obtained from 16S rRNA sequencing was analyzed using the Kruskal-Wallis test to compare the difference between two groups or all four treatments. *P* < 0.05 was considered significant.

## Results

### Serum 25-Hydroxycholecalciferol and Calcium Concentration

**Figure 1:**
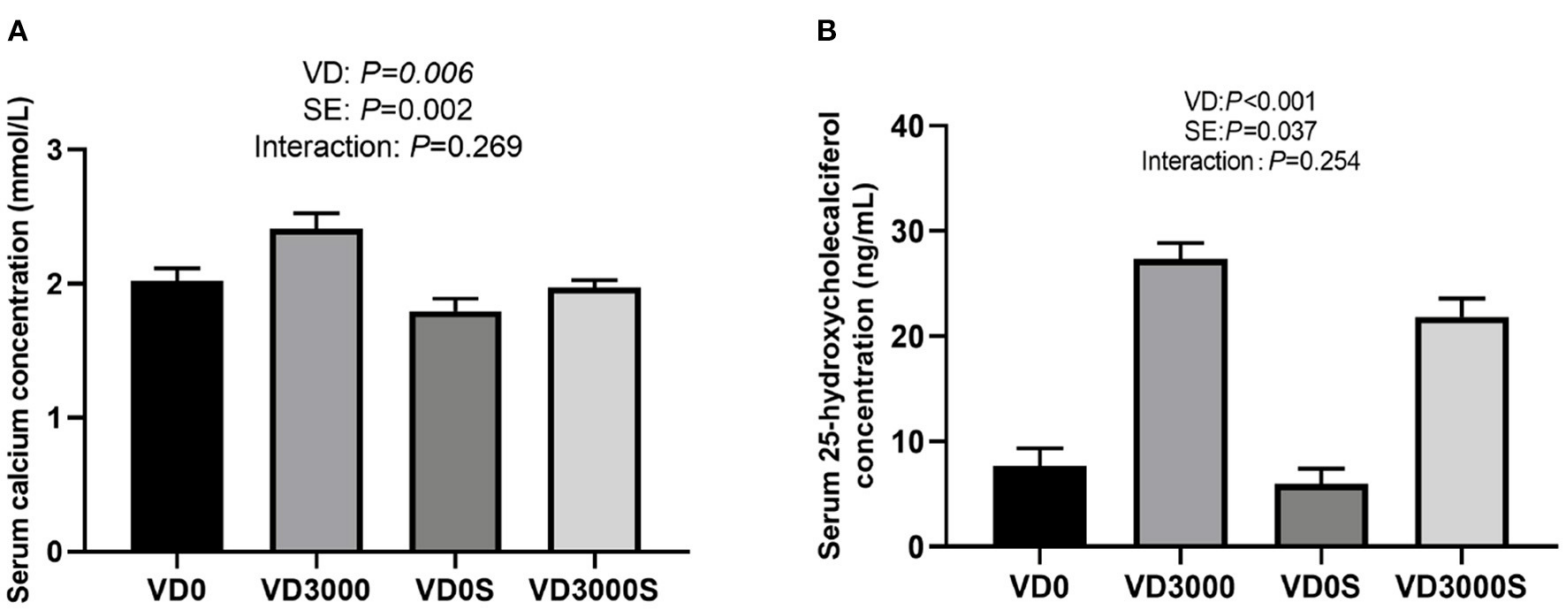
VD_3_ enhanced serum **(A)** calcium and **(B)** 25-OH-VD concentration of laying hens. Data are represented as the mean (*n* = 6) ± SEM; VD0, hens with VD_3_-free diet and no infection; VD3000, hens with VD_3_-sufficient diet and no infection; VD0S, hens with VD_3_-free diet and Salmonella infection; VD3000S, hens with VD_3_-sufficient diet and Salmonella infection; VD, vitamin D_3_; SE, *Salmonella enteritidis*.

Figure 1 showed that the concentrations of serum calcium/in VD_3_ sufficient groups (2.19 ± 0.09 mmol/L; 24.6 ± 1.37 ng/mL) were remarkably higher than those in VD_3_-deficient groups (1.91 ± 0.76 mmol/L; 6.76 ± 1.11 ng/mL) regardless of *Salmonella* infection (*P* = 0.006), indicating that hens fed diets without VD_3_ supplementation for 10 weeks were in a state of VD deficiency [deficiency <12 ng/ml; insufficiency was between 12 and 20 ng/ml (31)], whereas hens given VD_3_ at 3000IU/kg diet were VD sufficient. In addition, the *Salmonella* challenge reduced serum 25-OH-VD, and calcium (1.88 ± 0.06 mmol/L; 13.89 ± 2.63 ng/ml) concentration relative to the non-infected groups (*P* < 0.05, 2.22 ±0.09 mmol/L; 17.5 ± 3.16 ng/ml), suggesting that *Salmonella* challenge disturbed the absorption and metabolism of VD_3_ in hens, resulting in reduced serum calcium and VD levels. However, there was no notable interactive effect between VD_3_ addition and SE challenge on serum calcium and 25-OH-VD levels.

### Productive Performance

Data on the productive performance of hens fed diets with different levels of VD_3_ from 1 to 10 weeks before SE infection have been presented in our published paper (32). Data on the productive performance of hens fed diets with 0 or 3,000 IU VD_3_/kg of the diet with or without SE infection during 11 to 12 weeks was shown in this study (Table 2). Results indicated that supplemental VD_3_ at 3,000 IU per kg of diet significantly increased laying rate, egg weight, and feed intake remarkably improved feed to egg ratio (*P* < 0.01) but had no effect on mortality when compared with the VD_3_-free groups (*P* = 0.441). *Salmonella* challenge significantly decreased laying rate and egg weight (*P* < 0.05), trended to decrease the feed intake (*P* = 0.095), but notably increased the feed to egg ratio and death ratio (*P* < 0.05). There were interactive effects between VD_3_ addition and *Salmonella* challenge on egg weight as well as feed intake (*P* < 0.05). SE-infected hens without VD_3_ supplementation exhibited the lowest egg weight and feed intake compared with the other three groups. The non-infected hens fed VD-deficient diets showed the lowest egg thickness and eggshell strength as compared to the other three group hens. And there was an interactive trend of VD_3_ and challenge on feed egg ratio (*P* = 0.063). SE-infected hens fed VD_3_-deficient diets displayed the highest FCR relative to the other three group hens.

**Table 2:** VD_3_ improved the productive performance of *Salmonella* infected hens.

**Items**	**Dietary VD**_3_ **levels, IU/kg**				**SEM**	***P*** **-values**		
	**0**		**3000**			
	**Saline**	**SE**	**Saline**	**SE**		**VD_**3**_**	**SE**	**Interaction**
Laying rate, %	42.05	21.66	94.68	84.25	7.95	<0.001	0.003	0.257
Egg weight, g	60.78^a^	54.17^b^	61.74^a^	60.10^a^	0.84	0.001	<0.001	0.009
Feed egg ratio	4.11^b^	7.53^a^	1.86^b^	2.32^b^	0.66	<0.001	0.020	0.063
Feed intake, g	96.76^c^	78.67^d^	108.33^b^	116.90^a^	3.86	<0.001	0.095	<0.001
Death ratio, %	0.00	9.03	0.00	11.40	1.50	0.441	<0.001	0.441

*Data are presented as mean (n = 6) ± SEM. Means within a row lacking a common superscript differ significantly (P < 0.05). SEM, standard error mean. VD_3_, vitamin D_3_; SE, Salmonella enteritidis*.

### Intestinal Histopathological Changes

**Figure 2:**
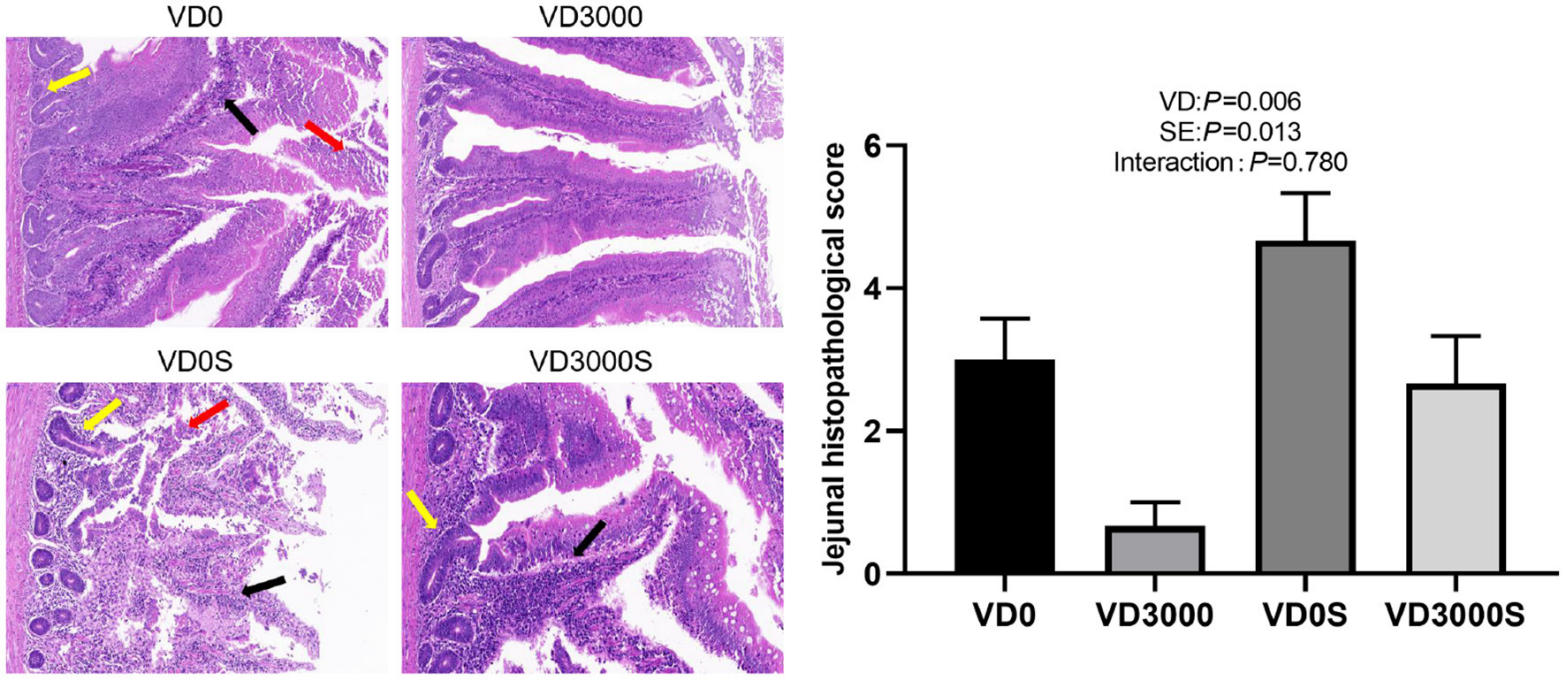
VD_3_ alleviated jejunal pathology in *Salmonella* infected hens (× 400 magnification; scale bar: 20 μm). Data are represented as the mean (*n* = 6) ± SEM; The photomicrographs showed obvious inflammatory-cell infiltration (black arrows), ulceration of the epithelial layer, necrotic cell clusters with shrunken nucleoli or karyorrhexis and karyolysis (red arrows), and connective tissue hyperplasia and inflammatory cell infiltration in the submucosal and muscle layer (crypt damage, yellow arrows). VD0, hens with VD_3_-free diet and no infection; VD3000, hens with VD_3_-sufficient diet and no infection; VD0S, hens with VD_3_-free diet and *Salmonella* infection; VD3000S, hens with VD_3_-sufficient diet and *Salmonella* infection; VD, vitamin D_3_; SE, *Salmonella enteritidis*.

Histopathological scores in the jejunum were detected by H&E (Figure 2). Results showed the jejunal morphology was destroyed by SE challenge and VD_3_ deficiency (*P* < 0.05). Compared with the VD_3_ sufficiency and non-*Salmonella* infected group, both the single *Salmonella*-infected hens and the VD_3_-deficiency hens showed extensive granulocytes and mono-nuclear cells infiltration in the mucosa and submucosa, ulceration of the epithelial layer, crypt damage of gut wall in the jejunum, resulting in substantially higher histopathology scores, indicating that either *Salmonella* infection or VD_3_ deficiency-induced intestinal mucosa impairment and intestinal inflammation. In contrast, only mild inflammation in the jejunum was observed in the infected hens that received VD_3_ sufficient supplementation compared to that of the single *Salmonella* infection hens, suggesting that dietary VD_3_ sufficient supplementation might alleviate intestinal inflammatory injury.

### *Salmonella* Load in the Cecal Digesta

**Figure 3:**
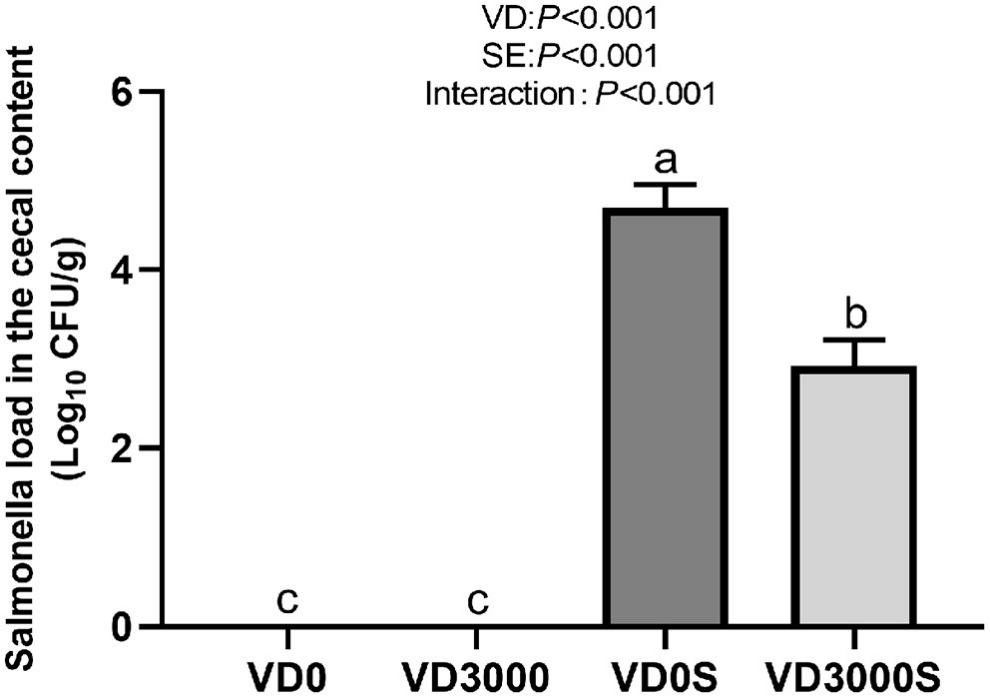
VD_3_ lowered *Salmonella* load in cecal content of *Salmonella* infected hens. Data are represented as the mean (*n* = 6) ± SEM; Bars with different letters show a significant difference between groups (*P* < 0.05); VD0, hens with VD_3_-free diet and no infection; VD3000, hens with VD_3_-sufficient diet and no infection; VD0S, hens with VD_3_-free diet and *Salmonella* infection; VD3000S, hens with VD_3_-sufficient diet and *Salmonella* infection; VD, vitamin D_3_; SE, *Salmonella enteritidis*.

As shown in Figure 3, no *Salmonella* was detected in the cecum of the two non-infected groups. Dietary VD_3_ addition down-regulated the number of *Salmonella* gene copies in cecal content nevertheless SE challenge up-regulated it significantly (*P* < 0.001). Infected hens given VD_3_ sufficient supplementation significantly decreased *Salmonella* numbers in cecal content compared with infected hens, indicating that supplemental VD_3_ could inhibit the growth of *Salmonella* in the cecum of infected hens (*P* < 0.001).

### Intestinal Cells' Apoptosis and Apoptosis-Related Proteins Abundances

**Figure 4:**
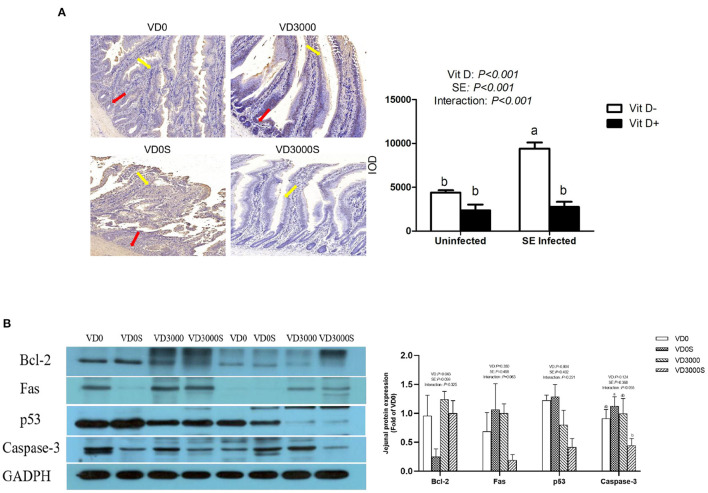
VD_3_ suppressed jejunal apoptosis response of *Salmonella* infected hens. **(A)** Percentage of TdT-mediated dUTP nick-End labeling (TUNEL)-positive cells. The red arrow points apoptotic cells in the jejunal crypt, and the yellow arrow represents the apoptotic cells in the villus. Integral optical density (IOD) represented the TUNEL expression. Magnification = 400×. **(B)** Western blot analysis for the expression levels of apoptosis-relative protein. Data are represented as the mean (*n* = 6) ± SEM; Bars with different letters show a significant difference between groups (*P* < 0.05); VD0, hens with VD_3_-free diet and no infection; VD3000, hens with VD_3_-sufficient diet and no infection; VD0S, hens with VD_3_-free diet and *Salmonella* infection; VD3000S, hens with VD_3_-sufficient diet and *Salmonella* infection; VD, vitamin D_3_; SE, *Salmonella enteritidis*; Bcl-2, B-cell lymphoma-2; GAPDH, glyceraldehyde-3-phosphate dehydrogenase.

Apoptotic cells were identified by TUNEL staining. The apoptotic cell had round and shrunken shapes and the nuclei were darkly stained. As illustrated in Figure 4A, VD sufficiency significantly decreased the TUNEL-positive cells population compared with VD deficiency (*P* < 0.001), nevertheless TUNEL-positive cells in the jejunum markedly increased in the single SE-infected hens compared with the non-infected group (*P* < 0.001). In addition, a marked reduction of TUNEL-positive cells was observed in the jejunum of the SE-infected hens given sufficient VD_3_ supplementation (*P* < 0.05). Western blotting results showed that SE infection tended to decrease the Bcl-2 protein transcription (*P* = 0.059), while VD sufficiency significantly increased anti-apoptosis Bcl-2 protein expression abundance, notably lowering the protein amount of p53 (*P* < 0.05, Figure 4B). Furthermore, relative to the infected hens without VD_3_ addition, infected hens given VD_3_-sufficient diets notably decreased Caspase-3 protein levels in the jejunum mucosa (*P* < 0.05). Based on these results, we suggested that dietary VD_3_ sufficient supplementation could attenuate *Salmonella*-induced intestinal cells apoptosis through modulating apoptosis-related proteins expression.

### Vitamin D_3_ Metabolism and Immune-Related Genes Expressions

As displayed in Table 3, *Salmonella* infection didn't affect intestinal VD-metabolism related genes expression, but remarkably upregulated toll-like receptor (TLR)-4, tumor necrosis factor (TNF)-α, interleukin (IL)-8, and interferon (IFN)-γ genes expression (*P* < 0.05), tended to up-regulate genes expressions of myeloid differentiation primary response protein 88 (MyD88) and IL-1β (0.05 < *P* < 0.1) as compared to the non-infected hens, suggesting that *Salmonella* infection caused intestinal inflammation *via* activating TLR4-mediated pathway. Supplemental VD_3_ significantly upregulated cytochrome P450 family 24 subfamilies A member 1 (CYP24A1), 25-hydroxylase-encoding gene cytochrome P450 family 2 subfamily R member 1 (CYP2R1), and TLR-2 genes expressions (*P* < 0.05), tended to upregulate 1α-hydroxylase-encoding gene cytochrome P450 family 24 subfamily B member 1 (CYP27B1), and cytochrome P450 family 3 subfamily A member 37 (CYP3A37) gene expressions (.05 < *P* < 0.1), and notably downregulated TNF-α mRNA levels (*P* = 0.053) and showed a decreased trend for TLR-4 and MyD88, and VDR gene expression in the jejunum (0.05 < *P* < 0.1) compared with the VD_3_-deficient hens, indicating that VD deficiency could induce intestinal inflammation, whereas intestinal inflammation induced by VD deficiency could be inhibited by VD sufficiency. SE-infected hens given the VD_3_-sufficient diets markedly upregulated jejunal CYP27B1 and TLR-2 but downregulated TLR-4, MyD88, IFN-γ, TNF-α, IL-1β as well as IL-8 mRNA levels (*P* < 0.05).

**Table 3:** VD_3_ facilitated VD_3_ metabolism, regulated inflammatory pathway and strengthened barrier function of jejunum of *Salmonella* infected hens.

**Items**	**Dietary VD**_3_ **levels, IU/kg**				**SEM**	***P-*** **values**		
	**0**		**3000**			
	**Saline**	**SE**	**Saline**	**SE**		**VD_**3**_**	**SE**	**Interaction**
**VD**_**3**_ **metabolism-related genes**								
VDR	1.57	1.21	1.03	0.95	0.11	0.070	0.251	0.624
RXR	1.27	1.21	1.06	1.63	0.11	0.819	0.377	0.142
CYP24A1	0.32	0.63	0.60	0.63	0.07	0.049	0.922	0.167
CYP2R1	0.40	0.30	1.03	0.81	0.09	0.000	0.130	0.538
CYP27B1	1.36^ab^	0.63^b^	1.01^ab^	2.00^a^	0.21	0.077	0.421	0.006
CYP3A37	0.32	0.33	0.61	0.55	0.07	0.089	0.862	0.807
**Immune-related genes**								
TLR-2	1.09^a^	0.43^b^	1.11^a^	1.49^a^	0.13	0.010	0.284	0.011
TLR-4	0.66	0.42	1.02	0.74	0.09	0.081	0.002	0.024
MyD88	0.99^ab^	0.73^b^	1.07^ab^	1.66^a^	0.13	0.075	0.052	0.048
NF-κB	0.94	0.84	1.02	1.11	0.05	0.866	0.765	0.283
IFN-γ	0.40	0.43	1.04	1.24	0.15	0.138	<0.001	0.024
TNF-α	0.82	0.74	1.00	2.06	0.17	0.035	0.002	0.006
IL-1β	0.23	0.35	1.14	1.03	0.15	0.661	0.085	0.038
IL-8	0.68	0.29	1.13	1.10	0.14	0.106	0.049	0.025
**Tight junction protein genes**								
Claudin-1	0.59	0.80	1.04	1.14	0.10	0.048	0.573	0.702
Occludin	3.04^ab^	1.35^b^	1.06^b^	4.55^a^	0.52	0.794	0.423	0.002
ZO-1	1.61^a^	1.25^b^	1.02^b^	1.51^a^	0.10	0.488	0.890	0.036
Mucin-2	2.59	2.05	1.03	1.39	0.27	0.039	0.849	0.366

*Data are presented as mean (n = 6) ± SEM. Means within a row lacking a common superscript differ significantly (P < 0.05). SEM, standard error mean. VD3, vitamin D3; SE, Salmonella enteritidis; VDR, vitamin D receptor; RXR, retinoid X receptor; CYP24A1, cytochrome P450 family 24 subfamily A member 1; CYP2R1, cytochrome P450 family 2 subfamily R member 1; CYP27B1, cytochrome P450 family 24 subfamily B member 1; CYP3A37, cytochrome P450 family 3 subfamily A member 37; TLR-2, toll-like receptor-2; TLR-4, toll-like receptor-4; MyD88, myeloid differentiation factor 88; NF-κb, nuclear factor kappa-B; IFN-γ, interferon-γ; TNF-α, tumor necrosis factor-α; IL-1β, interleukin-1β; IL-8, interleukin-8, ZO-1, zonula occludens-1*.

### Tight Junction Related Genes and Proteins Expressions

**Figure 5:**
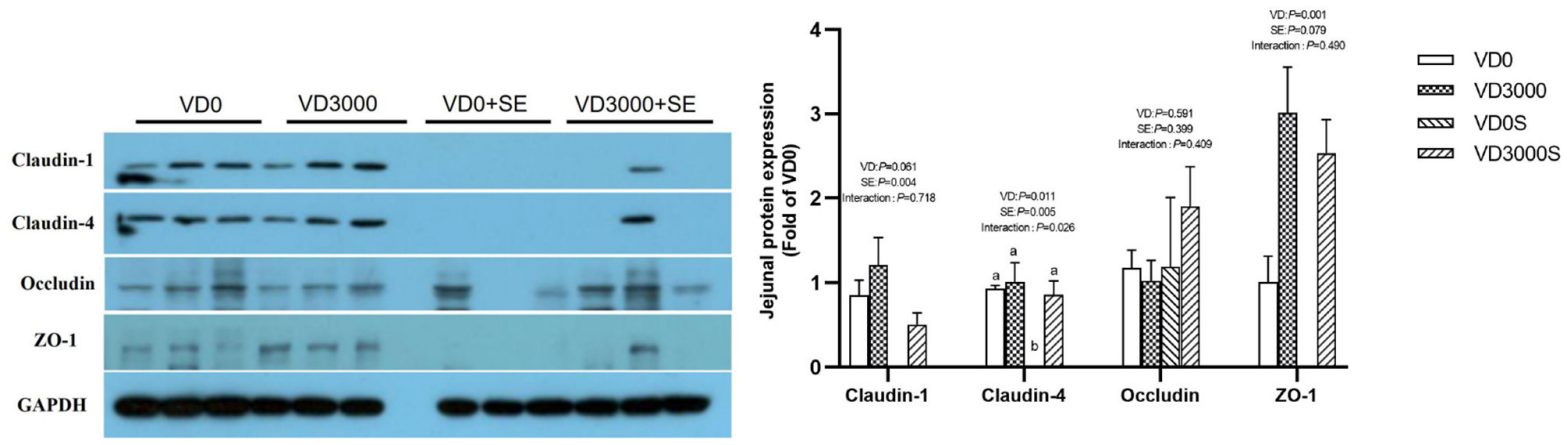
VD_3_ boosted jejunal barrier integrity of *Salmonella* infected hens. Data are represented as the mean (*n* = 6) ± SEM; Bars with different letters show a significant difference between groups (*P* < 0.05); VD0, hens with VD_3_-free diet and no infection; VD3000, hens with VD_3_-sufficient diet and no infection; VD0S, hens with VD_3_-free diet and *Salmonella* infection; VD3000S, hens with VD_3_-sufficient diet and *Salmonella* infection; VD, vitamin D_3_; SE, *Salmonella enteritidis*; ZO-1, zonula occludens-1; GAPDH, glyceraldehyde-3-phosphate dehydrogenase; VD, vitamin D_3_; SE, *Salmonella enteritidis*.

Compared with VD_3_-deficient groups, Claudin-1 gene expression was up-regulated by dietary VD_3_ sufficient supplementation, but mucin-2 was down-regulated (*P* < 0.05, Table 3). Although the SE challenge had no influence on the barrier function-related genes and mucin-2 expression, there were notable interactive effects on Occludin and ZO-1 mRNA expression between VD_3_ administration and *Salmonella* challenge (*P* < 0.05). Further analysis revealed SE-infected hens given VD_3_ sufficient addition displayed a remarkable increase in Occludin and ZO-1 mRNA levels compared with the SE-infected alone hens (*P* < 0.05). In accordance with mRNA expression results, immune-blotting showed that SE challenge significantly reduced the amount of Claudin-1 and Claudin-4 protein (*P* < 0.05, Figure 5), showed a declined trend in ZO-1 protein amounts (*P* = 0.079), indicating SE infection disrupted intestinal tight junction. In contrast, VD_3_ sufficient addition remarkably up-regulated the protein levels of Claudin-4 as well as ZO-1 (*P* < 0.05) and trended to increase the Claudin-1 protein amount (*P* = 0.061) regardless of infection. Further, SE-infected hens received VD sufficiency markedly upregulated Claudin-4 protein expression level, indicating a barrier restoring effect of VD_3_ sufficient addition on laying hens under the SE challenge.

### Cecal Microbiome

**Figure 6:**
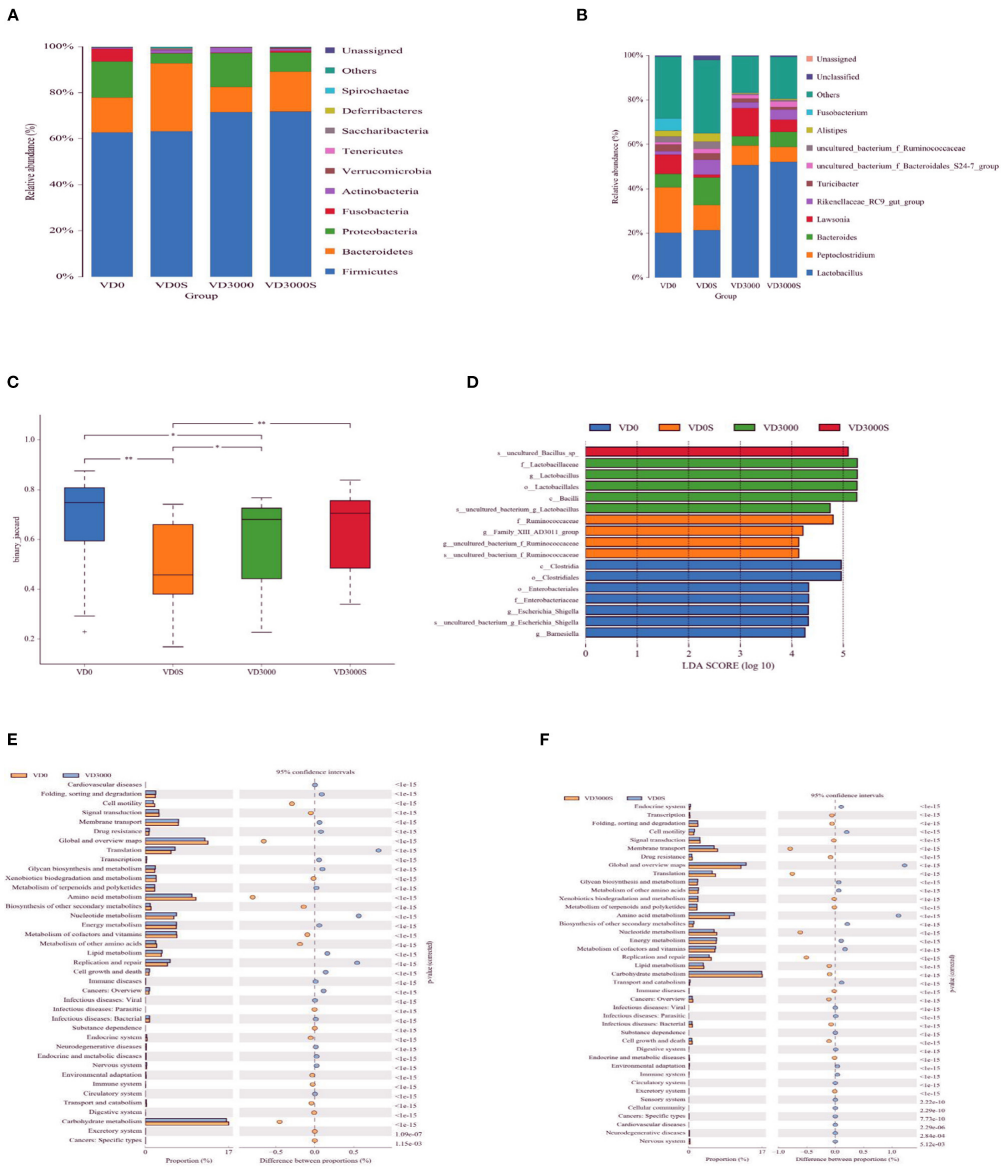
VD_3_ increased the abundance of fecal *Lactobacillus* to alleviated intestinal damage of *Salmonella* infected hens. Relative abundance of cecal microbiota at the **(A)** phylum level **(B)** geneus level. **(C)** Differential cecum microbiota community (β-diversity) between groups. **(D)** Differential enrichment of cecal microbiota between groups measured using LEfSe (LDA score = 4). The microbial pathways grouped into level-2 functional categories using PICRUSt between groups **(E)** VD0 and VD3000 and **(F)** VD3000 and VD3000S. Data are represented as the mean (*n* = 6) ± SEM; *P < 0.05, **P < 0.01. VD0, hens with VD_3_-free diet and no infection; VD3000, hens with VD_3_-sufficient diet and no infection; VD0S, hens with VD_3_-free diet and *Salmonella* infection; VD3000S, hens with VD_3_-sufficient diet and *Salmonella* infection.

The analysis of the 16S rRNA sequence showed that the cecal microbiota was prominently dominated by *Firmicutes, Bacteroidetes, Proteobacteria, Fusobacteria* together with *Actinobacteria* at the phylum level among all groups (Figure 6A). VD_3_ treatment had no influence on the relative abundance of those phyla, but the SE challenge remarkably decreased the abundance of *Proteobacteria* (*P* = 0.039, Table 4). What's more, dietary VD_3_ sufficient inclusion tended to inhibit SE-induced a decrease in the relative abundance of *Fusobacteria* compared to the single SE challenge (*P* = 0.079). At the gena level, microflora was dominated by *Lactobacillus, Peptoclostridium, Bacteroides, Lawsonia* as well as the *Rikenellaceae*_*RC9*_*gut*_*group* (Figure 6B). Analysis noted that SE infection tended to decrease *Lawsonia* (*P* = 0.086) but increased *Rikenellaceae_RC9_gut_group* relative abundance, respectively (*P* = 0.098, Table 4). Dietary VD_3_ sufficient supplementation enriched the abundance of *Lactobacillus* (*P* = 0.006). Our results also found that alpha diversity of cecal microflora was not affected by VD_3_ nutritional status and *Salmonella* challenge (*P* > 0.05, Table 5). However, a significant difference in β diversity was observed between groups VD0, VD0S, and VD3000 (*P* < 0.05, Figure 6C). Additionally, the β diversity of the VD3000S group was significantly different from the group VD0S (*P* < 0.001). Linear discriminant analysis coupled with effect size measurements (LEfSe) analysis showed that *Barnesiella, Escherichia Shigella, Enterobacteriaceae, Enterobacteriales, Clostridiales*, and *Clostridia* were rather abundant in the VD0 group (Figure 6D). Adding VD_3_ at 3,000 IU/kg enriched the abundance of *Lactobacillus, Lactobacillaceae, Lactobacillales* as well as *Bacilli*. SE infection up-regulated the abundance of *Ruminococcaceae, Family X AD3011*. Nevertheless, SE infected given VD_3_ sufficient addition enhanced an uncultured *Bacillus* abundance in cecal content compared with infected hens and the VD_3_-free hens. PICRUSt analysis indicated that sufficient supplementation with VD_3_ up-regulated the translation, nucleotide metabolism as well as replication and repair pathways, whereas it down-regulated cell motility, global and overview maps, amino acid metabolism, and carbohydrate metabolism pathways of cecal microbiota (*P* < 0.001, Figure 6E), when compared with VD0 group. Similarly, under the SE challenge, dietary VD_3_ increased the membrane transport, translation, nucleotide metabolism, replication, and repair pathways, but decreased amino acid metabolism pathways of cecal microbiota in level 2 (*P* < 0.001, Figure 6F).

**Table 4:** Changes in the composition of the gut microbiota cecal microbiota of laying hens challenged with *Salmonella* (%).

**Items**	**Dietary VD**_3_ **Levels, IU/kg**				**SEM**	***P-*** **values**		
	**0**		**3000**					
	**Saline**	**SE**	**Saline**	**SE**		**VD_**3**_**	**SE**	**Interaction**
**Phylum level**								
*Firmicutes*	60.15	61.18	72.20	72.13	3.85	0.154	0.952	0.945
*Bacteroidetes*	21.02	32.84	10.72	18.06	3.85	0.109	0.217	0.770
*Proteobacteria*	13.88	3.43	14.11	7.52	2.03	0.588	0.039	0.627
*Actinobacteria*	3.94	0.10	0.23	0.59	0.96	0.412	0.376	0.288
*Fusobacteria*	0.45	0.92	2.41	0.80	0.30	0.121	0.329	0.079
**Genera level**								
*Lactobacillus*	16.35	22.39	50.44	48.71	5.54	0.006	0.835	0.708
*Peptoclostridium*	16.16	9.35	9.13	8.86	2.50	0.472	0.497	0.531
*Bacteroides*	7.05	13.37	4.13	7.48	1.68	0.195	0.157	0.660
*Lawsonia*	7.49	0.86	11.95	4.62	1.99	0.304	0.086	0.930
*Rikenellaceae_RC9* *_gut_group*	1.42	7.05	2.66	4.54	1.11	0.773	0.098	0.400

*Data are presented as mean (n = 6) ± SEM. Means within a row lacking a common superscript differ significantly (P < 0.05). SEM, standard error mean. VD_3_, vitamin D_3_; SE, Salmonella enteritidis*.

**Table 5:** Changes in the diversity of the gut microbiota cecal microbiota of laying hens challenged with *Salmonella*.

**Items**	**Dietary VD**_3_ **Levels, IU/kg**				**SEM**	***P-*** **values**		
	**0**		**3000**			
	**Saline**	**SE**	**Saline**	**SE**		**VD_**3**_**	**SE**	**Interaction**
ACE	241.01	330.10	290.17	300.51	15.11	0.742	0.102	0.191
Chao1	241.33	335.27	286.79	292.87	15.93	0.961	0.119	0.169
Simpson	0.19	0.11	0.23	0.22	0.02	0.116	0.322	0.463
Shannon	2.69	3.51	2.56	2.76	0.17	0.203	0.143	0.368

*Data are presented as mean (n = 6) ± SEM. Means within a row lacking a common superscript differ. VD_3_, vitamin D_3_; SE, Salmonella enteritidis*.

## Discussion

In this study, we first investigated the changes in the productive performance of laying hens at different VD_3_-metabolic statues (deficient or sufficient) with or without SE infection. A significant decrease in egg production and egg weight, and a notable increase in feed to egg ratio, were observed in hens that received a VD_3_-free diet, indicating that dietary long-term VD_3_ deficiency had significant negative effects on the productive performance of laying hens. However, these changes in productive performance caused by VD deficiency were reversed by dietary VD_3_ sufficient addition, which was consistent with previous reports (32, 33). Additionally, data of this study also showed that *Salmonella* infection had adverse effects on productive performance of laying hens which were similar to previous studies, while these negative effects caused by SE challenge were lightened by VD_3_ sufficient supplementation (3,000 IU per kg of diet), suggesting that dietary VD_3_ sufficient supplementation could reverse the negative impact of productive performance caused by *Salmonella* infection or VD deficiency in laying hens.

The major circulating form of VD is the 25-OH-VD, whose serum levels are regarded as an indicator of VD metabolic status, availability, and biological functions (34). In the study, we observed that serum Ca and 25-OH-VD concentration were notably decreased by *Salmonella* challenge or VD_3_ insufficiency or both treatments, reflecting that either *Salmonella* challenge or dietary VD_3_ deficiency treatment or both could reduce calcium and VD_3_ absorption in the gut, thereby resulting in intestinal malabsorption syndromes and VD deficiency status. In contrast, feeding sufficient VD_3_ significantly reversed this trend caused by *Salmonella* challenge or dietary VD_3_ deficiency, suggesting that dietary VD_3_ sufficient supplementation could improve the VD nutritional status of hens that were exposed to either *Salmonella* challenge or dietary VD_3_ deficiency. VD_3_ exerts various biological functions, mainly through regulating VD metabolic enzymes 25-hydroxylase (CYP2R1), 1α-hydroxylase (CYP27B1), and CYP24A1 activity, along with the VDR signal pathway (35, 36). Meanwhile, results of RT-PCR analysis showed that VD_3_ sufficient supplementation upregulated CYP2R1 and CYP24A1 genes expressions, tended to upregulate CYP27B1 and CYP3A37 gene expressions, showing dietary VD_3_ sufficiency facilitated the metabolic pathway of VD, resulting in increasing serum 25-OH-VD level (36). Thus, we suggested that changes in productive performance caused by dietary VD_3_ levels and *Salmonella* infection were possibly related to Ca and 25-OH-VD contents in serum. Dietary addition of sufficient VD_3_ contributed to the improvement of production performance in our study was partially ascribed to the observed higher serum calcium and 25-OH-VD concentration and upregulated intestinal CYP24A1, CYP2R1, and CYP27B1 genes expression.

A plethora of studies has demonstrated that VD3 and VDR signal pathways play a pivotal role in intestinal homeostasis through modulating intestinal barrier function (TJs and AJs), microbiome composition, as well as immune responses. Moreover, undesirable VD nutritional status or loss of VDR has long been associated with intestinal barrier impairment, dysbiosis, inflammation, and disease in humans and mice (21, 37). However, the effect of VD nutritional status on the intestinal health of *Salmonella*-infected hens has been rarely reported. In this study, we further investigated the effect of VD nutritional status on intestinal cells apoptosis index, intestinal pathological scores, and cecal *Salmonella* load of the *Salmonella*-infected hens. Results indicated that SE challenge or VD_3_ insufficiency or both increased intestinal histopathological scores, the number of intestinal TUNEL-positive cells together with *Salmonella* colonization levels, while VD_3_ sufficiency reversed these alterations caused by *Salmonella* infection or VD_3_ insufficiency, as indicated by a reduction in *Salmonella* carrier, TUNEL-positive cells percentage as well as gut inflammation, suggesting that VD_3_ deficiency promoted overgrowth of *Salmonella* in inflamed gut, increased the susceptibility to *Salmonella* infection, deteriorated intestinal damage severity. While VD_3_ sufficiency could restrain *Salmonella* proliferation and colonization in the gut together with decrease intestinal inflammation and apoptosis, resulting in ameliorating gut damage caused by *Salmonella* infection or VD deficiency. Similar results were also reported by previous research using mice or humans as models (38, 39), indicating that VD_3_ nutritional status is associated with disease susceptibility and the degree of gut injury.

Intestinal epithelial cells' apical junctional proteins, including Claudins, Occludins, ZOs, junctional adhesion molecules (JAMs), and E-cadherins, play a vital role in regulating intestinal permeability, defense against pathogen infection, and inflammation response (40). TLR-mediated signaling pathways and apoptosis response play core regulative roles in responding to *Salmonella* infection and epithelial barrier integrity (27, 41). In order to reveal the correlation between VD_3_ nutritional status and gut health of the *Salmonella*-infected or VD-deficient hens in deep, intestinal mucosal immune-related genes, apoptosis-related proteins, and tight junction proteins expressions profiles were analyzed further. In this study, *Salmonella* infection activated TLR4-mediated immune responses, induced pro-inflammatory cytokines and chemokines expression (IFN-γ, TNF-α, IL-1β, and IL-8), and suppressed intestinal tight junction Claudin-1, Claudin-4 protein, and ZO-1 protein amounts in laying hens, which was in consistent with previous results using chicken models (3, 42). Meanwhile, the protein amount of pro-apoptotic p53 and caspase-3 in the jejunum was elevated by the SE challenge. Additionally, the study also found that the effects of VD_3_ deficiency on intestinal immune responses, intestinal cells apoptosis, and tight junction proteins expression of hens were similar to that of the *Salmonella* challenge. Based on the above changes, we suggested that SE infection or VD deficiency, or both treatments lead to intestinal inflammation and intestinal epithelial cells apoptosis, possibly resulting in elevated intestinal mucosal permeability and the impairment of gut barrier integrity in laying hens. In contrast, those alterations in the gut induced by *Salmonella* infection or VD_3_ deficiency were restrained by VD_3_ sufficient administration in our study, as evidenced by downregulating TLR-mediated inflammatory cytokines (TLR4, MyD88, and TNF-a) genes expressions, upregulating Claudin-1 gene and protein, Claudin-4 and ZO-1 protein abundances in the VD-sufficient hens irrespective of *Salmonella* infection relative to VD deficiency. Moreover, upregulation in Claudin-4 protein, anti-apoptosis protein Bcl-2 amount, and VDR, Occludin as well as ZO-1 mRNA levels; together with downregulation in TLR-4, MyD88, IFN-γ, TNF-α, IL-1β, and IL-8 mRNA levels in the gut of the *Salmonella*-infected hens given enough VD_3_ obtained in our study, suggesting that VD_3_ sufficiency can effectively prevent intestinal inflammation and intestinal epithelial cells apoptosis triggered by SE infection, and protected intestinal epithelial barrier integrity. Similar results have been observed in humans and mice when faced with stress and pathological conditions (38). Meanwhile, VD_3_ sufficient supply enhanced TLR-2 mRNA levels in *Salmonella*-infected hens compared with the infected hens with VD insufficiency was detected in our study. VDR and TLR-2 signal pathways had been reported to be positively involved in anti-infection, anti-inflammatory, anti-oxidative and anti-apoptosis activities as well as intestinal barrier-protective effects, especially under inflammation conditions (43), therefore upregulated level of TLR-2 together with intestinal VDR mRNA amounts obtained in *Salmonella*-infected hens received enough VD_3_, indicating further confirmed our suggestion. Collectively, our results suggested that VD_3_ sufficient supply could prevent intestinal barrier impairment induced by either *Salmonella* or VD deficiency, and improve gut health in laying hens, possibly by reducing intestinal mucosal inflammation and boosting tight junction proteins expression together with inhibiting intestinal epithelial cells apoptosis. Our data obtained from the present study also suggests that VD_3_ sufficient supply may be beneficial for controlling *Salmonella* infection in laying hens.

Intestinal microbiota and its metabolites play a critical role in regulating host physiology, immune responses, and diseases resistance ability (44). Evidence from mammal experimental studies and clinical trials suggested that there were potential associations between VD_3_, gut microbiota, and gut health, namely, the effect of VD_3_ on health is partially mediated through the microbiome (45, 46). To investigate the underlying mechanisms of the VD_3_ status on the gut health of the *Salmonella*-infected hens, the cecal microbial composition was further investigated. The results revealed that the alpha diversity of the microbiota profile was not different between the VD_3_ nutritional status and *Salmonella* challenge in our study, which was similar to the results of research on humans and mice (47). However, both VD_3_ treatment and SE challenge remarkably modified β-diversity, which indicated that VD_3_ administration, SE challenge altered intestinal bacterial community profiles. LEfSe analysis observed that some potential detrimental microbes, including *Barnesiella, Escherichia Shigella, Enterobacteriaceae, Enterobacteriales, Clostridiales*, and *Clostridia*, were enriched in VD_3_-deficient hens, while sufficient VD_3_ sufficient addition favored the growth of some potential health-beneficial microbes, including *Lactobacillales, Lactobacillaceae*, and *Lactobacillus*, as well as *Bacilli*, implying that VD nutritional status was correlated with gut microbiome composition in laying hens irrespective of *Salmonella* infection. In similar with our findings, accumulating pieces of evidence from human and mice studies has shown that the composition of the gut microbiome can be altered by VD_3_ status, especially under disease challenge conditions, and the effects of VD_3_ on intestinal microbiome interactions related to gut dysbiosis and gut inflammation (48). In mammals, supplemental VD_3_ could increase the abundance of potential health-beneficial bacterial genera (such as *Lactobacillus*), and lowered the abundance of potential detrimental or inflammation-related microbiota genera in the gut, possibly by regulating the production of antimicrobial peptides, such as cathelicidin and β-defensin (49). Additionally, VD deficiency increases the relative abundance of opportunistic pathogens which, in the context of intestinal barrier dysfunction, may favor pathogen bacterial translocation and systemic infection and inflammation (50). *Lactobacillus* spp. was reported to exhibit beneficial effects on intestinal health, and intestinal barrier integrity, and had stronger anti-inflammatory effects when confronted with pathogens infection, including *Salmonella* (51). Also, *Lactobacillus* treatment could contribute to the increase in circulating VD, improve the VDR signaling and reduce the inflammatory response in the gut (52, 53). Higher serum 25-OH-VD levels decreased intestinal inflammation and improved gut barrier structure observed in the *Salmonella*-infected or VD_3_-deficient hens following sufficient VD_3_ supplementation in our study might be attributed to the increase of *Latcobacillus* abundance in the gut. At the same time, VD_3_ sufficient addition enriched uncultured *Bacillus* abundance in the cecal content of the *Salmonella*-infected given as compared to the single infected hens and the VD_3_-free hens. Some *Bacillus* spp were considered to be beneficial microbiota and used as feed additives and antibiotic alternatives due to secreting digestive enzymes, producing anti-microbial substances along with possessing immune-regulatory and anti-inflammatory effects (54). Further, more studies had showed that probiotics *Bacillus* spp. was widely used to advance feed absorption and productive performance, promote immunity, improve gut health and enhance resistance to enteric pathogens, such as *Salmonella, Escherichia coli, and Clostridium perfringens*, infection in poultry (3). Collectively, these findings suggested VD nutritional status affects the composition of gut bacterial microbiota, and VD deficiency could bring about gut dysbiosis and gut barrier integrity impairment, while supplementation of sufficient VD_3_ had a positive influence on health-beneficial microbes in the *Salmonella*-infected hens or the VD_3_-deficient hens. In other words, the protective effect of VD_3_ sufficiency on the gut health of the *Salmonella*-infected hens was possibly by promoting the establishment of a healthy microbiota and inhibiting potential detrimental microbes. Improved laying performance, decreased mortality, and alleviated gut inflammation observed in hens supplied with sufficient VD_3_ may be possibly attributed to improving gut microbial composition induced by VD_3_ sufficient addition. PICRUSt analysis indicated that nucleotide metabolism, translation as well as replication, and repair pathways involved in cell viability and growth were enriched in the hens that received VD_3_ sufficient addition regardless of *Salmonella* infection (55). Meanwhile, the membrane transport pathway associated with the survival of bacteria in the gut ecosystem was also increased in the *Salmonella*-infected hens given sufficient VD_3_, suggesting that VD_3_ sufficient supplementation exerted a growth-promoting, restorative role for gut microflora of hens under an undesirable state of nutrition or pathogen challenge (56). Improved intestinal microbiota in hens following VD_3_ sufficient administration whether infected with *Salmonella* or not was obtained in our study to support our suggestion. In addition, cell motility and amino acid metabolism pathways were suppressed by dietary VD_3_ sufficiency relative to VD deficiency. Further, only amino acid metabolism pathways were downregulated in the *Salmonella*-infected hens given sufficient VD_3_ as compared to the single *Salmonella*-infected hens. Cell motility is the determinant step of pathogen bacteria, such as *Salmonella*, in early local invasion (57). The unabsorbed amino acids in the hind-gut would be metabolized into polyamines, such as putrescine, spermidine, and spermine, which could damage the gut (58). Thus, the down-regulated cell motility and amino acid metabolism pathways suggested that VD_3_ might have anti-infective and barrier-protecting functions *via* modulating the function of gut microbiota. Surprisingly, carbohydrate metabolism pathways were enriched in the VD_3_-deficient group. As reported, indigestible carbohydrate could be fermented by cecal microflora into short-chain fatty acids which functions as trophic and immune regulatory effects (59). Combined with the phenomenon of potential harmful microbiota, such as *Barnesiella, Escherichia Shigella, Enterobacteriaceae, Enterobacteriales, Clostridiales*, and *Clostridia*, were abundant in VD_3_-deficient hens, we suggested that carbohydrate metabolism pathways enriched in VD_3_-deficient hens may be associated with the production of inflammatory metabolites and intestinal inflammation. Taken together, observation in the present study indicated that VD_3_ sufficient supplementation might be a valuable way to manipulate the composition of the bacterial microbiome and protect against gastrointestinal injury in hens. Additional studies are needed to further understand the relationship between VD_3_ levels, gut bacteria, and gut health using fecal microbiota transplantation.

## Conclusion

In summary, we presented evidence that the VD_3_ status is associated with gut health and disease resistance of laying hens. Concretely speaking, VD_3_ insufficiency or deficiency are much more susceptible to *Salmonella*, leading to intestinal microflora disturbance and gut mucosal injury together with extensive intestinal inflammatory responses in laying hens. While dietary supplementation with sufficient VD_3_ is able to enhance intestinal barrier junctions by inducing junction protein expression, restoring the balance of intestinal microbiota, and alleviating intestinal damage and intestinal inflammation induced by *Salmonella* in laying hens. The data also first identified that adequate VD_3_ in feed could act as a novel nutritional strategy to defend against *Salmonella* infection in hens. It should be noted that well-defined studies on optimal dose and underlying action mechanisms of VD_3_ modulating gut health of hens are required in future research.

## Data Availability Statement

The datasets presented in this study can be found in online repositories. The names of the repository/repositories and accession number(s) can be found below: NCBI [accession: PRJNA759703].

## Ethics Statement

The animal study was reviewed and approved by CAU20170601-2.

## Author Contributions

ZW designed the research, evaluated the test details, and revised the manuscript. YqG and WA performed the experiments. FG analyzed the data and wrote the manuscript. WZ, SW, and YH participated in the revision of the manuscript. YmG and QM provided calculation and operation support. All authors contributed to data interpretation and approved the final version of the manuscript.

## Funding

This research was funded by the Natural Science Foundation of China (No. 32172774).

## Conflict of Interest

The authors declare that the research was conducted in the absence of any commercial or financial relationships that could be construed as a potential conflict of interest.

## Publisher's Note

All claims expressed in this article are solely those of the authors and do not necessarily represent those of their affiliated organizations, or those of the publisher, the editors and the reviewers. Any product that may be evaluated in this article, or claim that may be made by its manufacturer, is not guaranteed or endorsed by the publisher.

## Supplementary Material

Figure S1 Action mechanisms of VD3 modulating gut health of hens with VD3 deficiency and/or *Salmonella* challenge: VD3 sufficiency ameliorated gut injury induced by either *Salmonella* and/or VD3 deficiency via promoting VD3 metabolism, reducing *Salmonella* load, suppressing pro-inflammatory status, increasing expression of TJs along with decreasing apoptotic responses. Besides, VD3 restored the balance of gut microflora by enriching the abundance of probiotics such as *Lactobacillus* and *Bacilli*.
