# Supplementary material for: Vitamin D3 Nutritional Status Affects Gut Health of Salmonella-Challenged Laying Hens
Source: Front Nutr. 2022 May 10;9:888580. doi: 10.3389/fnut.2022.888580 (PMC9127613; doi:10.3389/fnut.2022.888580)
Supplement: Supplementary file 1 [file Table_1.docx]

Supplementary Material

# Supplementary Tables

**TABLE S1. Sequences of primers for quantitative real-time PCR**

| Target genes | Primer sequence (5'-3') | Accession NO. |
| --- | --- | --- |
| β-actin | F: GAGAAATTGTGCGTGACATCA  R: CCTGAACCTCTCATTGCCA | L08165 |
| VDR | F: AGAGGAAAGCGATGTTCACC  R: CCTTCATCATCCCAATGTCC | XM_015272607.1 |
| RXR | F: GATGCGAGACATGCAGATG  R: CGGGGTATTTGTGCTTG | XM_015279790.1 |
| CYP24A1 | F: AACATTTCACGCAATCCACA  R: AATGGCACAGATGGTGTCAA | XM_015296334.1 |
| CYP2R1 | F: CATCATAGCGGGGACGGAAA  R: CTGCAACCGGATGAGCAGAT | XM_004941395.2 |
| CYP27B1 | F: CAAAGGAGACATCGTGGA  R: TCGTGGGTAAATAGGCTTC | NM_204976.2 |
| CYP3A37 | F: CGAATCCCAGAAATCAGA  R: AGCCAGGTAACCAAGTGT | NM_001001751.2 |
| Claudin-1 | F: TGGAGGATGACCAGGTGAAGA  R: CGAGCCACTCTGTTGCCATA | NM_001013611.2 |
| Occludin | F: TCATCGCCTCCATCGTCTAC  R: TCTTACTGCGCGTCTTCTGG | NM_205128.1 |
| ZO-1 | F: TATGAAGATCGTGCGCCTCC  R: GAGGTCTGCCATCGTAGCTC | XM_413773.4 |
| Mucin-2 | F: TTCATGATGCCTGCTCTTGTG  R:CCTGAGCCTTGGTACATTCTTGT | NM_001318434.1 |

*F, forward; R, reverse. Primers were synthesized by Sango Biotech (Shanghai) Co., Ltd.*

*VDR, vitamin D receptor; RXR, retinoid X receptor; CYP24A1, cytochrome P450 family 24 subfamily A member 1; CYP2R1, cytochrome P450 family 2 subfamily R member 1; CYP27B1, cytochrome P450 family 24 subfamily B member 1; CYP3A37, cytochrome P450 family 3 subfamily A member 37. ZO-1, zonula occludens-1.*

**TABLE S2.** Sequences of primers for quantitative real-time PCR

| Target genes | Primer sequence (5'-3') | Accession NO. |
| --- | --- | --- |
| TLR-2 | F: ACCTTCTGCACTCTGCCATT  R: TGTGAATGAAGCACCGGTAA | NM_204278.1 |
| TLR-4 | F: CCACTATTCGGTTGGTGGAC  R: ACAGCTTCTCAGCAGGCAAT | NM_001030693.1 |
| MyD88 | F: GGATGGTGGTCGTCATTTCA  R: GAGATTTTGCCAGTCTTGTCCA | NM_001030962.1 |
| NF-κB | F: ACCCCTTCAATGTGCCAATG  R: TCAGCCCAGAAACGAACCTC | NM_205129.1 |
| IFN-γ | F: AAAGCCGCACATCAAACACA  R: GCCATCAGGAAGGTTGTTTTTC | NM_205149.1 |
| TNF-α | F: CCCCTACCCTGTCCCACAA  R: TGAGTACTGCGGAGGGTTCAT | NM204267.1 |
| IL-1β | F: CAGCAGCCTCAGCGAAGAG  R: CTGTGGTGTGCTCAGAATCCA | NM_204524.1 |
| IL-8 | F: GGCTTGCTAGGGGAAATGA  R:AGCTGACTCTGACTAGGAAACTGT | NM_205498.1 |

*F, forward; R, reverse；Primers were synthesized by Sango Biotech (Shanghai) Co., Ltd. TLR-2, toll-like receptor-2; TLR-4, toll-like receptor-4; MyD88, myeloid differentiation primary response protein 88; NF-κB, nuclear factor kappa-B; IFN-γ, interferon-γ; TNF-α, tumor necrosis factor-α; IL-1β, interleukin-1β; IL-8, interleukin-8.*

**TABLE S3.** Primary antibodies used for immune blotting

| Protein | MW/KDa | Company | Cat No. | Dilution |
| --- | --- | --- | --- | --- |
| Bcl-2 | 26 | Wanlei Biotech Inc | WL03681 | 1:500 |
| Fas | 48 | Wanlei Biotech Inc | WL0030 | 1:5000 |
| p53 | 53 | Biodragon | BD-pm3380 | 1:2000 |
| Caspase-3 | 32 | Biodragon | BD-pm3435 | 1:500 |
| GAPDH | 37 | Biodragon | B1030 | 1:2000 |
| Claudin-1 | 22 | Self-prepared | - | 1:1000 |
| Claudin-4 | 22 | Self-prepared | - | 1:1000 |
| Occludin | 65 | Self-prepared | - | 1:1000 |
| ZO-1 | 130/225 | Self-prepared | - | 1:2000 |
