# Supplementary figures and images for: Vitamin D3 Nutritional Status Affects Gut Health of Salmonella-Challenged Laying Hens
Source: Front Nutr. 2022 May 10;9:888580. doi: 10.3389/fnut.2022.888580 (PMC9127613; doi:10.3389/fnut.2022.888580)

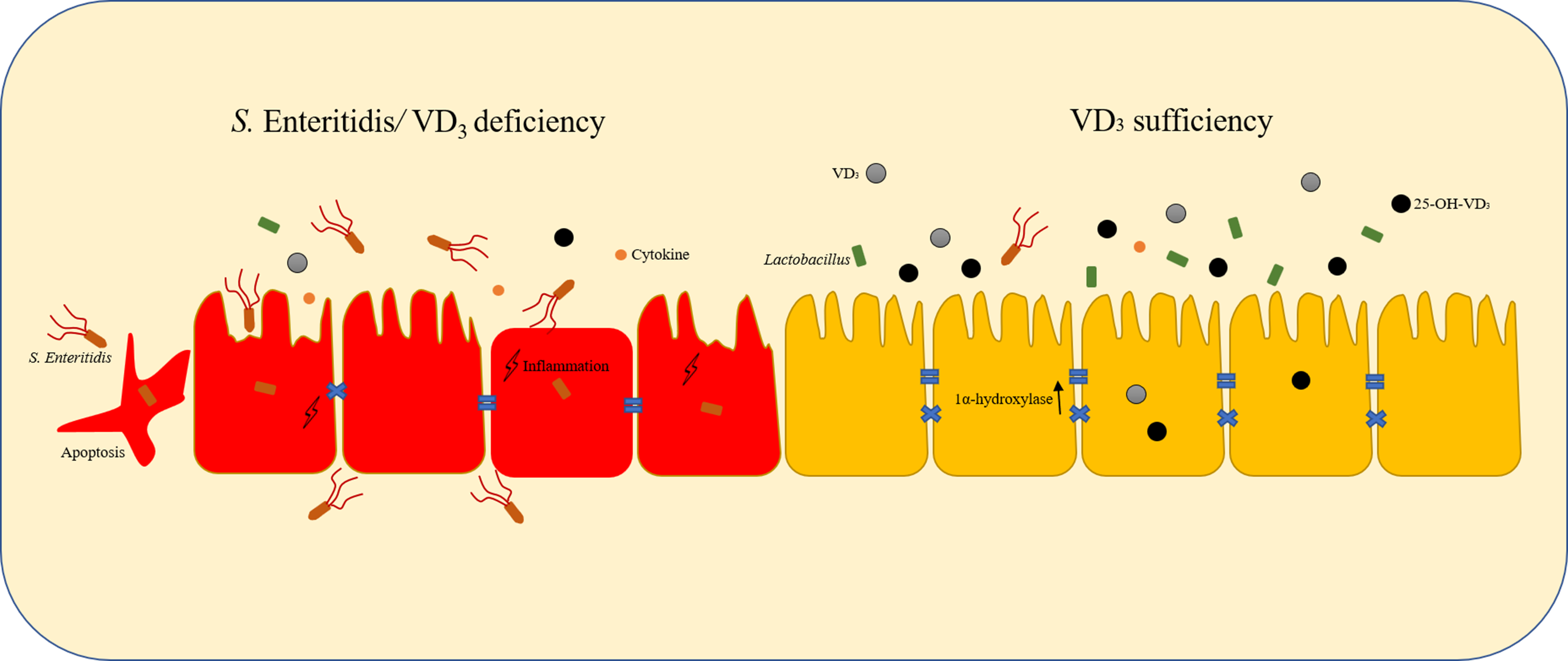

Supplement: Figure S1 — Action mechanisms of VD3 modulating gut health of hens with VD3 deficiency and/or Salmonella challenge: VD3 sufficiency ameliorated gut injury induced by either Salmonella and/or VD3 deficiency via promoting VD3 metabolism, reducing Salmonella load, suppressing pro-inflammatory status, increasing expression of TJs along with decreasing apoptotic responses. Besides, VD3 restored the balance of gut microflora by enriching the abundance of probiotics such as Lactobacillus and Bacilli. [file Image_1.PNG]
